# Comparison of IPAQ-SF and Two Other Physical Activity Questionnaires with Accelerometer in Adolescent Boys

**DOI:** 10.1371/journal.pone.0169527

**Published:** 2017-01-05

**Authors:** Triin Rääsk, Jarek Mäestu, Evelin Lätt, Jaak Jürimäe, Toivo Jürimäe, Uku Vainik, Kenn Konstabel

**Affiliations:** 1 Institute of Sport Sciences and Physiotherapy, Faculty of Medicine, University of Tartu, Tartu, Estonia; 2 Department of Psychology, University of Tartu, Tartu, Estonia; 3 National Institute for Health Development, Tallinn, Estonia; Vanderbilt University, UNITED STATES

## Abstract

Self-report measures of physical activity (PA) are easy to use and popular but their reliability is often questioned. Therefore, the general aim of the present study was to investigate the association of PA questionnaires with accelerometer derived PA, in a sample of adolescent boys. In total, 191 pubertal boys (mean age 14.0 years) completed three self-report questionnaires and wore an accelerometer (ActiGraph GT1M) for 7 consecutive days. The PA questionnaires were: International Physical Activity Questionnaire-Short Form (IPAQ-SF), Tartu Physical Activity Questionnaire (TPAQ), and the Inactivity subscale from Domain-Specific Impulsivity (DSI) scale. All three questionnaires were significantly correlated with accelerometer derived MVPA: the correlations were 0.31 for the IPAQ-SF MVPA, 0.34 for the TPAQ MVPA and -0.29 for the DSI Inactivity scale. Nevertheless, none of the questionnaires can be used as a reliable individual-level estimate of MVPA in male adolescents. The boys underreported their MVPA in IPAQ-SF as compared to accelerometer-derived MVPA (respective averages 43 and 56 minutes); underreporting was more marked in active boys with average daily MVPA at least 60 minutes, and was not significant in less active boys. Conversely, MVPA index from TPAQ overestimated the MVPA in less active boys but underestimated it in more active boys. The sedentary time reported in IPAQ-SF was an underestimate as compared to accelerometer-derived sedentary time (averages 519 and 545 minutes, respectively).

## Introduction

Self-report methods for assessing physical activity (PA) include retrospective questionnaires, interview-administered recall, activity diaries and mail surveys [1]. Questionnaires have been most commonly used to assess children’s subjective rating of PA, because of being relatively inexpensive and easy to use in large-scale studies [2–3]. However, self-report questionnaires have been shown to have weak to modest correspondence to objectively measured PA [4–6]. In contrast, accelerometers are found to be reliable and valid objective instruments for measuring PA in children and adolescents, but more expensive and time-consuming to use than self-report methods [6–8].

Objective and subjective methods assess PA in different ways. The criterion validity of The International Physical Activity Questionnaire (IPAQ) is usually assessed using accelerometers; with both instruments, one can use minutes per day in different intensity categories (e.g., moderate or sedentary) as unit [2,9,10]. Comparing absolute values, one has to take into account that accelerometer data reduction methods have shown large influence on the comparison of objective and subjective methods [1,10,11]. Meta-analysis results have shown small-to medium correlations between IPAQ and objective measurement [12], but in a few studies, no or very low correlations have been found [13–14].

In previous papers we have compared a moderate to vigorous physical activity (MVPA) index derived from Tartu Physical Activity Questionnaire (TPAQ, a self-report questionnaire of PA) with accelerometer derived MVPA in adolescent boys [15–16]. We have found that TPAQ-derived MVPA index was weakly associated with accelerometer-derived MVPA [16].

The differences between self-reported and accelerometer-measured PA are found to be larger at higher activity and intensity levels [11,12,17]. In addition, people do not have a built-in mechanism for telling moderate from vigorous activity, and the subjective boundary may considerably vary from person to person. The convergent correlations could thus be expected to be stronger for MVPA than for moderate PA (MPA) or vigorous PA (VPA) taken separately. In addition, international PA recommendations for adolescents are mostly about MVPA [18]. Our focus in this paper will therefore also be MVPA.

Adults and adolescents tend to overreport their PA in IPAQ, as compared to accelerometer measures [2,19,20]. Adolescents tend to overreport VPA and underreport their MPA in IPAQ short form (IPAQ-SF) [1,10,21]. In one study [22], however, underreporting of MPA and VPA was found in adolescents. The problem in that study may have been inaccurate criterion rather than underreporting: using ActiReg, they had weekly (7-day) average values of MPA and VPA as 845 and 256 minutes, respectively, which amounts to an unusually high level of 157 minutes of daily MVPA; using IPAQ, the comparable value was 55 minutes which is more realistic in European adolescents.

As the reliability of self-report measures of PA in adolescents is being questioned, there is a clear need of studies comparing questionnaires to objective criteria. Therefore, the general aim of the present study was to investigate the association of PA questionnaires with accelerometer-derived (ActiGraph GT1M) PA in a sample of 13-14-years-old pubertal boys. More specifically, we will examine both relative agreement (correlations) and absolute agreement (e.g., over- and underestimation) between the respective methods.

## Materials and Methods

### Participants and study design

This study was a part of the project “Risk factors for metabolic syndrome in boys during pubertal development: A longitudinal study with special attention to PA and fitness”. This project started in 2009 when all boys from 3^th^ and 4^th^ grades from Tartu, Estonia and its immediate surroundings were invited to participate [16,23,24]. The project invitation was then given to each boy of particular classes in those schools who agreed to participate and approximately 84% of them agreed to take part. Initial exclusion criteria were different health problems that did not allow the potential subjects to participate in physical education classes [15,25]. All participants were thoroughly informed of the purposes and contents of the project and a written informed consent was obtained from the parents before participation in the project. The participants gave verbal assent. All participants had the right to stop participating at any time-point of the project. This project was approved by the Medical Ethics Committee of the University of Tartu, Estonia.

This project had for four waves of data collection. After the first testing in the laboratory the participants were called back in the following three years. All four waves of data collections took place at approximately same time of the year: the first wave of data collection lasted from November 2009 to April 2010, the second wave from November 2010 to April 2011, the third wave from November 2011 to April 2012, and the fourth wave from November 2012 to April 2013.

In the present paper, we use data from the fourth data collection wave. The total sample size was 217, but due to missing data, the sample available for our analyses was 191 boys (age 14.0 ± 0.7 years).

During the research day, all participants completed self-report PA questionnaires: IPAQ-SF, TPAQ, and Domain-Specific Impulsivity (DSI) Inactivity scale. At the end of the research day, participants were asked to wear an ActiGraph GT1M accelerometer for seven consecutive days.

### Measurement of body parameters

Measurements of all body parameters were conducted by trained researchers. Participants’ body height and mass were measured on the first day of the measurements. Body height was measured in standing position to the nearest 0.1 cm using Martin metal anthropometer. Body mass was measured with minimal clothing with a medical balance scale (A&D Instruments, UK) to the nearest 0.05 kg. Body mass index (BMI) was calculated as the body mass divided by the square of body height (kg/m^2^).

### Objective measurement of PA

PA was assessed by accelerometer GT1M ActiGraph (Monrovia, CA, USA). The accelerometer has been previously validated in laboratory and free-living conditions in young people [26–28]. The accelerometer is compact, small (3.8x3.7x1.8cm) and light-weight (27g) uniaxial monitor designed to detect vertical accelerations ranging in magnitude from 0.05 to 2.00 G’s with a frequency of 0.25–2.50 Hz and converts the signal to numeric values known as activity counts [2,26]. We used the same intensity categories as previously used in other similar studies [27–29]: sedentary time (< 100 counts per min), light PA (LPA) (> 100 counts per min), MPA (> 2000 counts per min) and VPA (> 4000 counts per min). Additionally, MVPA was calculated by summing the highest two intensity categories. At the end of the research day, participants were asked to wear the accelerometer always on the right hip with adjustable elastic belt for 7 consecutive days during waking hours, except during water and bathing activities. The participants received the accelerometer together with written and verbal instructions, and were demonstrated how to wear the device. The accelerometer was programmed to record activity counts in 15-s epochs; non-wear time (excluded from the analysis) was defined as ≥ 20 consecutive minutes of 0 counts. PA data were included for further analyses if the subject had accumulated a minimum of 8-hours of activity data per day for at least 3 days [27]. Raw data were downloaded into the ActiLife software (version 5.3.0) computer software and then imported to R [30] for further analysis. To avoid confounding by wear time, the variables referring to time in activity categories were transformed to “adjusted minutes” [31] by dividing the raw minutes by wear time and multiplying the resulting fraction by the average wear time.

### Subjective assessment of PA

The subjective assessments of PA included the IPAQ-SF, TPAQ, and DSI Inactivity scale (assessing the tendency to avoid being physically active). The self-report questionnaires were filled out during the research day and with the assistance of researchers.

IPAQ-SF is a self-report questionnaire that assesses PA in the last 7 days [9–10]. Using the IPAQ-SF scoring system, the total number of days and minutes of PA were calculated for each participant as recommended in the IPAQ website [32]. The IPAQ-SF records the activity in four intensity levels: sitting, walking, moderate intensity (e.g., leisure cycling), and vigorous intensity (e.g, running or aerobics). In this study we used sitting time, walking, and MVPA as converted to minutes per day.

TPAQ is a short self-report questionnaire that consists of 26 questions about the last week PA [16,33,34]. Nineteen of the questions were not directly about PA level (e.g., costs of attending the sports club; reasons for missing the physical education classes; does the respondent's best friend attend to a sports club, etc.). In our study, we only used the questions that were directly related to sedentary time, walking and MVPA; in some cases, information from two or more questions was combined to make up a single variable: (Question [Q]1) walking/cycling (minutes per week were computed by multiplying two answers: how many days in a typical week did the child go to school by foot or by bike, and how many minutes did it take; after this, weekly minutes of going to school and going home from school were added); (Q2) Sport club minutes per week (computed analogously to Q1); (Q3) Frequency of PA (how many days in the previous week was the child at least moderately active for at least 30 minutes); the score ranged from 0 to 7; (Q4) Frequency of PA yes vs. no (at least 30 minutes 5 times a week was the child moderately active); (Q5) Screen time (how many hours a day watching TV or using computer), coded as no time = “1”; less than 1 h = “2”; 2–3 h = “3”; 4–5 h = “4”; more than 6 h = “5”.

The TPAQ MVPA index was calculated as in a previous paper [15] using the formula derived from a multiple regression conducted with the data from the third wave of data collection of the same project: MVPA (logit transformed proportion) = -2.25476 + 0.00018*Q1 + 0.00023*Q2 + 0.01543*Q3 + 0.18694*Q4–0.05822*Q5. After this, the estimate was back transformed to proportion units using inverse logit, and then, for ease of presentation and comparability with objective MVPA, transformed to “adjusted minutes” by multiplying it by the average wear time. The rationale behind using the MVPA index is twofold. First, for comparing objective and subjective assessments of PA, we need to express both in a comparable way; that is, items from the questionnaire need to be combined into a single index. Secondly, the self-report questionnaire variables had different units (minutes per week, yes/no scale, number of days per week) which could not be combined by just adding all items. Using regression weights, we combined the information from all items in a concise way, and at the same time made the unit of the resulting index comparable with the objective assessment of MVPA.

As a measure of self-reported inactivity, we used the Inactivity subscale from the DSI scale (translated with the permission of the authors) [35]. That scale was developed to assess tendencies for short-term gratification at the expense of long-term goals and standards across six impulsivity domains, including exercise. The respondents were asked to rate how often they did each of the activities, on a scale from 0 = "Never" to 4 = "Very often". Sample items from the exercise subscale include "Avoiding working out (e.g., jogging, going to the gym, etc.)" and "Being sedentary". To the four original questions, we added "I prefer to move as little as possible". As the content of these as well as the other items in this subscale refer to being inactive or avoiding activity, we refer henceforth to this subscale as inactivity scale; the remaining DSI subscales were not used in the present study. The theoretical maximum of inactivity score was thus 20, but in reality, the options “often” and “very often” were seldom used, and almost half of the sample (47%) indicated a complete disagreement with all 5 items receiving thus a score of zero. 37% of the sample received a score between 1 and 5, and 16% had a score of 6 or higher.

### Statistical analyses

All analyses were made using R version 3.1.2 [30]. Descriptive statistics of the participants are presented as means ± standard deviation (SD), as well as quartiles. Differences between PA intensity categories measured by IPAQ-SF, TPAQ, DSI Inactivity scale and the accelerometer GT1M ActiGraph were examined by paired-sample t-test.

Pearson correlations were used to indicate the degree of association between variables. All variables with minute as measurement unit (i.e., accelerometer-measured time in intensity categories; subjective estimates of sedentary time, MPA and VPA from IPAQ-SF, as well as MVPA index from TPAQ) were log transformed before regression analyses (i.e., log transformation was used for correlations and multiple regression; the means and SDs were calculated with untransformed data and expressed in minutes).

Agreement between the questionnaires and accelerometer were also assessed using the Bland and Altman method [36] as implemented in R package MethComp version 1.22 [37].

## Results and Discussion

Descriptive statistics of the anthropometric variables, accelerometer derived PA, and self-reported PA in 13-14-years-old boys, are shown in Table 1.

**Table 1:** Descriptive statistics of the subjects (N = 191).

	Mean ± SD	25% / 50% / 75%
**Age (y)**	14.0 ± 0.7	13.2 / 14.0 / 14.9
**Body height (cm)**	169.2 ± 9.1	157.0 / 170.2 / 180.5
**Body mass (kg)**	60.5 ± 17.1	43.2 / 56.7 / 83.8
**BMI (kg/m^2^)**	20.9 ± 4.76	16.8 / 19.6 / 27.9
**Accelerometer measured PA**		
**Sedentary time (min/d)**	545.2 ± 56.1	475.7 / 542.9 / 616.7
**Light PA (min/d)**	167.7 ± 39.6	116.8 / 167.4 / 220.3
**Moderate PA (min/d)**	37.4 ± 15.7	20.8 / 36.1 / 56.8
**Vigorous PA (min/d)**	19.0 ± 14.7	4.6 / 14.6 / 39.0
**MVPA (min/d)**	56.4 ± 25.0	27.9 / 52.3 / 89.0
**Steps per day**	8022 ± 3010	4774 / 7307 / 12492
**Valid time (min/d)**	769.3 ± 108.9	619.5 / 783.0 / 889.5
**IPAQ-SF**		
**Sedentary time (min/d)**	519.2 ± 107.7	420.0 / 540.0 / 630.0
**Walking/cycling (min/d)**	21.3 ± 28.0	1.0 / 14.3 / 52.1
**Moderate PA (min/d)**	15.5 ± 15.4	0.0 / 12.9 / 34.3
**Vigorous PA (min/d)**	27.6 ± 23.7	2.8 / 20.4 / 60.0
**MVPA (min/d)**	43.0 ± 29.0	9.3 / 38.6 / 81.4
**TPAQ**		
**MVPA index (min/d)**	59.7 ± 9.1	48.1 / 61.4 / 70.4
**Walking/cycling (min/d)**	43.1 ± 40.4	0.0 / 250.0 / 750.0
**Screen time (1–5)**	3.3 ± 0.8	2.0 / 3.0 / 4.0
**DSI**		
**Inactivity subscale (0–20)**	2.5 ± 3.1	0.0 / 1.0 / 7.0

Body mass index (BMI); Domain-Specific Impulsivity (DSI); International Physical Activity Questionnaire-Short Form (IPAQ-SF); light physical activity (LPA); minutes per day (min/d); moderate physical activity (MPA); moderate to vigorous physical activity (MVPA); physical activity (PA); Tartu Physical Activity Questionnaire (TPAQ); vigorous physical activity (VPA); standard deviation (SD).

Note that IPAQ-based estimates of MVPA and sedentary time were somewhat smaller than the corresponding accelerometer-derived measures (respective averages were 43 and 56 minutes for MVPA, and 519 and 545 minutes for sedentary time).

Correlations between self-report questionnaires and accelerometer derived PA are shown in Table 2.

**Table 2:** Correlations between accelerometer and self-reported PA in 13-14-years-old boys.

	Accelerometer measured PA (min/d)					
	MVPA	VPA	MPA	LPA	Sedentary time	Steps
**TPAQ MVPA (min/d)**	0.35***	0.34***	0.27***	0.13	-0.22**	0.35***
**TPAQ screen time (min/d)**	-0.31***	-0.24***	-0.28***	-0.28***	0.31***	-0.30***
**TPAQ walking/cycling (min/d)**	0.21**	0.21**	0.16*	-0.02	-0.09	0.19**
**IPAQ MPA (min/d)**	0.16*	0.16*	0.11	0.07	-0.10	0.17*
**IPAQ VPA (min/d)**	0.31***	0.24***	0.29***	0.15*	-0.18*	0.27***
**IPAQ MVPA (min/d)**	0.31***	0.29***	0.25***	0.13	-0.16*	0.29***
**IPAQ walking (min/d)**	0.36***	0.34***	0.27***	0.07	-0.22**	0.32***
**IPAQ sedentary (min/d)**	-0.23**	-0.19*	-0.18*	-0.12	0.16*	-0.25***
**DSI Inactivity scale (min/d)**	-0.29**	-0.29***	-0.22*	-0.13	0.17*	-0.27**

Sample size for inactivity questionnaire was 129, and ranged from 183 to 190 for other questionnaires.

* p < 0.05

** p < 0.01

*** p < 0.00.

Domain-Specific Impulsivity (DSI); International Physical Activity Questionnaire-Short Form (IPAQ-SF); light physical activity (LPA); minutes per day (min/d); moderate physical activity (MPA); moderate to vigorous physical activity (MVPA); physical activity (PA); Tartu Physical Activity Questionnaire (TPAQ); vigorous physical activity (VPA).

All questionnaires were correlated with accelerometer-measured PA; the significant correlations ranged from 0.07 to 0.36 for IPAQ-SF, from 0.02 to 0.35 for TPAQ, and from 0.13 to 0.29 for the DSI Inactivity scale.

To find whether questionnaires under- or overestimated PA as compared to accelerometer-derived PA, we used paired t-tests. We found that IPAQ-SF underestimated MVPA (mean difference -13.4 minutes per day as compared to accelerometer; t (186) = -5.46, p < 0.0001). Underreporting was more marked in active boys, whose daily MVPA was at least 60 minutes (mean difference -31,5 minutes per day; t (72) = 8.3, p < 0.0001), and was not significant in less active boys (mean difference -1.9 minutes per day; t (113) = 0.68, p = 0.50). The IPAQ-SF walking, as well as TPAQ walking/cycling were gross underestimates of LPA as measured by the accelerometer (IPAQ walking: mean difference -146.1 minutes per day; t (187) = 1.81, p < 0.0001; TPAQ walking/cycling: mean difference -125.1 minutes per day; t (186) = -30.16, p < 0.0001). The IPAQ-SF walking time was, on the average, significantly lower than TPAQ walking/cycling time (mean difference 24.0 minutes per day; t (206) = 8.07, p < 0.0001). Finally, the IPAQ-SF sedentary time was an underestimate as compared to the accelerometer-derived sedentary time (mean difference -27.1 minutes per day; t (184) = -3.32, p = 0.001).

**Fig 1:**
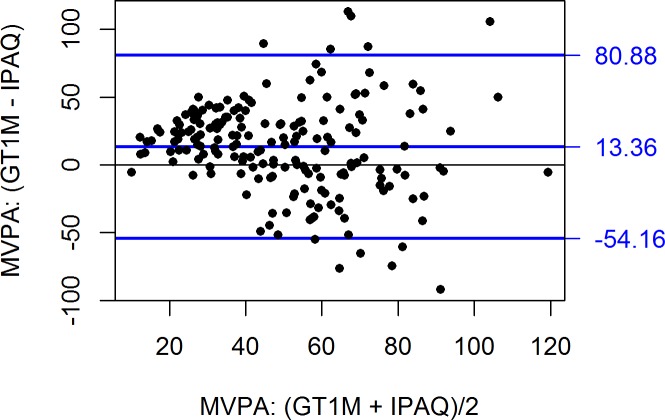
Bland and Altman plots with difference in mean time spent in MVPA for the IPAQ-SF and accelerometer GT1M ActiGraph. In the Bland and Altman plot, difference between two measurements is plotted against their mean. Mean and 95% confidence intervals of the difference are shown with blue lines. If there is no bias (in this case, over- or underestimating), then mean error should be close to zero. In addition, most measurement points should ideally be within the 95% confidence limits of the mean error (that is, within the lower and the upper blue line). International Physical Activity Questionnaire (IPAQ); moderate to vigorous physical activity (MVPA).

**Fig 2:**
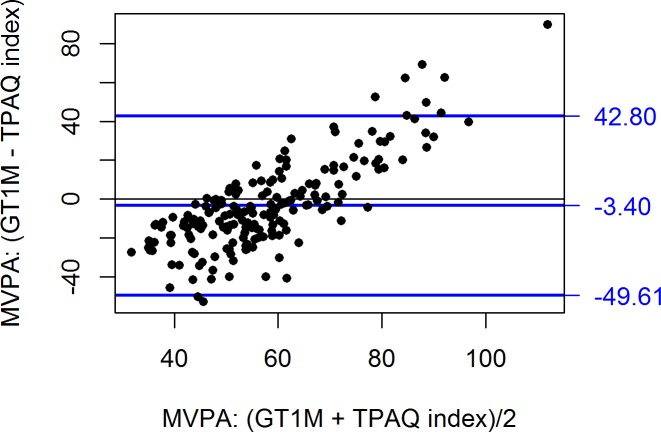
Bland and Altman plots with difference in mean time spent in MVPA for the TPAQ and accelerometer GT1M ActiGraph. Moderate to vigorous physical activity (MVPA); Tartu Physical Activity Questionnaire (TPAQ).

**Fig 3:**
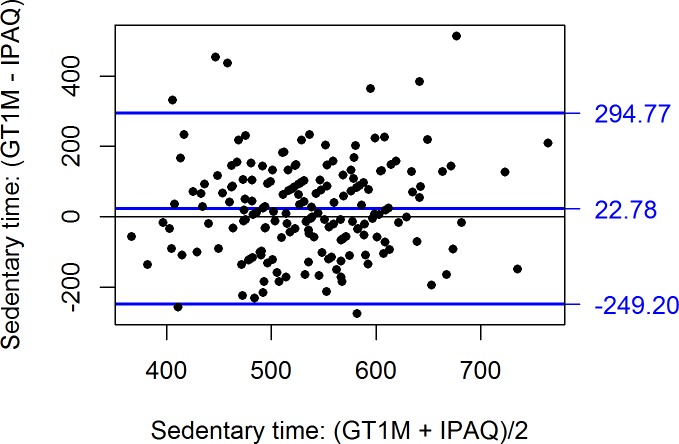
Bland and Altman plots with difference in mean time spent in sedentary time for the IPAQ-SF and accelerometer GT1M ActiGraph. International Physical Activity Questionnaire (IPAQ).

Agreement between the questionnaires and accelerometer was also assessed using the Bland and Altman method [36]. In Fig 1, one can see that the IPAQ-SF underestimated the MVPA slightly less at higher activity levels (r = -0.14, p < 0.05). In Fig 2, one can see a reverse but much stronger pattern for TPAQ MVPA index: overestimation at low activity levels but underestimation at higher activity levels (r = 0.78, p < 0.0001). For IPAQ sedentary time (Fig 3), accuracy was unrelated to activity level (r = -0.06, p = 0.40) but the prediction error was rather large (95% confidence limits from -249 to +295 minutes).

For finding an optimal prediction model of PA, we used multiple regressions. First, we regressed accelerometer MVPA on all variables derived from IPAQ-SF. The results are shown in Table 3. IPAQ-SF self-reported VPA and MPA, as well as walking and sedentary time were predictive of objective MVPA (all regression coefficients were statistically significant), even though the R-squared was modest (0.26). Adding TPAQ screen time to the prediction increased R-square to 0.29; in that model, TPAQ screen time was a significant predictor but IPAQ-SF sedentary time was not significant at 0.05 levels. Adding any other items from TPAQ, or the TPAQ MVPA index, or DSI Inactivity scale did not improve the prediction.

**Table 3:** Multiple regression models for predicting MVPA from IPAQ-SF variable.

	b	SE(b)	t	P
**Model 1**				
**(Intercept)**	5.81	1.00	5.83	< 0.0001
**IPAQ-SF VPA (min/d)**	0.09	0.03	3.39	0.0009
**IPAQ-SF MPA (min/d)**	0.06	0.03	2.08	0.0386
**IPAQ-SF walking (min/d)**	0.13	0.03	4.91	< 0.0001
**IPAQ-SF sedentary time (min/d)**	-0.41	0.16	-2.66	0.0086
**Model 2**				
**(Intercept)**	3.17	0.11	28.66	< 0.0001
**IPAQ-SF VPA (min/d)**	0.10	0.03	4.16	< 0.0001
**IPAQ-SF MPA (min/d)**	0.06	0.03	2.08	0.0394
**IPAQ-SF walking (min/d)**	0.13	0.03	5.02	< 0.0001

All variables were log transformed before analysis. Coefficient of determination (R^2^) for Model 1 was 0.26, N = 181, p < 0.0001 R^2^ for Model 2 was 0.23, N = 181, p < 0.0001. R^2^ was 0.11 for the model with only VPA and MPA as predictors.

International Physical Activity Questionnaire-Short Form (IPAQ-SF); moderate physical activity (MPA); minutes per day (min/d); probability level (p); regression weight (b); standard error of the regression weight [SE(b)]; value of the t-statistic (t); vigorous physical activity (VPA).

The best regression model using TPAQ items as predictors of MVPA included two significant predictors: TPAQ screen time and TPAQ walking/cycling (R^2^ = 0.16, N = 187, p < 0.0001). In contrast to our previous results [15], time in sports club was not predictive of MVPA when screen time was controlled for, even though there was a univariate correlation between MVPA and time in sports club. Adding DSI Inactivity scale to the model including TPAQ screen time and walking/cycling as predictors, increased the R^2^ to 0.19 (p < 0.0001).

TPAQ screen time was the only useful predictor of accelerometer measured LPA. The best model for predicting sedentary time included two predictors: TPAQ screen time and IPAQ-SF walking (R^2^ = 0.14). IPAQ-SF sedentary time was not significant in the model after controlling for screen time and walking.

### Summary of results

In this study we found that IPAQ-SF, TPAQ and DSI Inactivity scale were correlated with accelerometer measured PA in 13-14-years-old pubertal boys. MVPA estimates from IPAQ-SF and TPAQ, as well as DSI Inactivity scale were moderately correlated with accelerometer derived MVPA. Boys underreported their MVPA in IPAQ-SF; however, TPAQ MVPA index was an overestimate of MVPA compared to the accelerometer derived MVPA. None of the questionnaires can be used as a reliable estimate of MVPA at the individual level in pubertal boys.

### Agreement between self-reports and accelerometer derived PA

PA guidelines usually set a desired value in terms of daily or weekly minutes of PA—MPA, VPA, or MVPA. A common use of PA questionnaires is evaluating how well an individual or a group conforms to the guidelines. From the three above mentioned criteria, objective MVPA was best predicted by questionnaires, followed by VPA and MPA. One can speculate that VPA is easier to estimate subjectively, as it is usually means relatively short episodes of effort that are clearly separated from other activities (e.g., a game of basketball, or an hour of running). In contrast, the upper and lower thresholds of MPA, and the lower threshold of VPA are intuitively not as clear; thus, the total minutes in MPA, as well as VPA are more difficult to estimate in a questionnaire. In addition, self-estimated MVPA is, in most cases, a sum of two estimates, and can thus be more reliable than its individual components (random errors in each of the component may cancel each other out).

We also saw that both MPA and VPA, as well as MVPA was correlated with several variables derived from questionnaires, including sedentary time, screen time, and walking. For example, IPAQ-SF walking and sedentary time taken together contributed more to the prediction of MVPA than IPAQ-SF MPA and VPA. Here, one should take into account that there is no one-to-one correspondence between the activity categories in subjective estimates, and those in objective measures. For example, brisk walking may be MPA when measured objectively, but it classifies as walking in IPAQ-SF. The behaviours that are reported as walking in IPAQ-SF may include some LPA or possibly even below that threshold, but walking is definitely not the only activity in the LPA category. Thus, when estimating actual PA from questionnaires, it can be useful to take into account several different kinds of information that was recorded, not just the specific question about the intensity category that one wants to predict.

Sedentary time was more difficult to predict than PA; in the best regression model, it was predicted by screen time and walking (negative correlation). Inexactness of accelerometer derived sedentary time estimate may have contributed to this result: we could only use a heuristic approximation for separating sleeping and nonwear time from sedentary time. In addition, sedentary time is probably difficult to estimate subjectively, as it consists of many episodes dispersed throughout the day. The fact that a more specific questionnaire item (screen time) was a better predictor of sedentary time (r = 0.31) than an overall sedentary time estimate from IPAQ (r = 0.16) may reflect this cognitive difficulty.

The best predictor of LPA in our study was screen time—this was obviously a negative predictor. This makes sense considering that light and sedentary intensity activities together make up the largest proportion of time, thus if one of them increases, the other is likely to decrease. This “competition” between sedentary time and LPA can be seen in several longitudinal studies. For example, in 2-year-old boys in IDEFICS study [31] had, on the average, 400 daily minutes of LPA, and 200 minutes of sedentary time. At 10 years of age, however, they spent about 350 minutes in both sedentary time and LPA. 14-year-old boys in our study had, (Table 1) on the average day, 168 minutes of LPA, and 545 minutes of sedentary time. This dramatic decrease in LPA is likely to play a bigger role in energy balance than small changes in MVPA, and indicates that the importance of LPA may be underestimated in PA guidelines.

### Accuracy of self-reports of PA

The PA recommendation is at least 60 minutes MVPA per day [18]. If questionnaires are used to evaluate the conformance to guidelines, we need accuracy in addition to mere correlation agreement. We found that boys underreported their MVPA in IPAQ-SF as compared to accelerometer derived MVPA (respective averages 43 and 56 minutes). This is in opposition with another study where overreporting was found (respective average 66 and 34 minutes) [13].

Our study confirmed the finding that differences between self-reported and accelerometer derived MVPA are higher with higher activity levels [11,12,17]; this result makes sense as more active participants probably have a more varied pattern of activities, and, as a result, the total volume of MVPA is more difficult to estimate. We also found that to be true for bias: underestimation in self-reported MVPA was only significant in active boys (MVPA > = 60 minutes). This reduces the conflict with earlier studies which found overestimation for IPAQ: for example, in one study [13] the mean daily MVPA was 34 minutes, compared to 56 minutes in our study.

The TPAQ MVPA index slightly overestimated accelerometer derived MVPA; the likely reason for this is that the index was calibrated against data from a previous (more physically active) year.

IPAQ-SF walking cannot be treated as an estimate of LPA—this would be an immense underestimation of about 147 daily minutes. In IPAQ-SF, the respondents are instructed to count only the episodes of walking lasting at least 10 minutes—this may be one source of discrepancy. On the other hand, LPA includes several different activities besides walking (e.g., many leisure-time activities), that may be difficult to count and ask in a questionnaire.

IPAQ-SF underestimated sedentary time compared to accelerometer derived sedentary time (mean difference about 25 minutes). Taken together with low intercorrelation (r = 0.19), this indicates that IPAQ-SF sedentary time is not a reliable estimate of actual sedentary time, even though the mean differences might not be large.

### Using self-report questionnaires to estimate PA

Overall, none of the questionnaires used in the present study can be considered as a reliable individual-level estimate of PA in male adolescents. Questionnaires can, however, provide some information potentially usable for group comparisons or for examining the correlates of PA. Based on our results, we would recommend using all IPAQ-SF items, not just the sum of MPA and VPA, to predict MVPA. IPAQ-SF walking and sedentary time contributed more to the prediction than the two PA variables. IPAQ-SF walking cannot be treated as an estimate of LPA—first, it probably includes some MPA, and second, most LPA are not included.

The unique contribution of TPAQ was mostly confined to the screen time item; other information may be derived from IPAQ-SF. The TPAQ MVPA index, based on a linear combination of 5 different variables, appears a reasonable predictor of MVPA if only the size of correlation is considered. However, the bias of TPAQ MVPA index was linearly dependent on PA (Fig 2): the index overestimated PA at low levels of PA but underestimated it at high levels of PA. The TPAQ MVPA index is thus unsuitable for individual level prediction, even though it might provide a good approximation of a group’s average PA. One has to take into account also the fact that in our study, TPAQ was self-administered, whereas assistance was provided with IPAQ-SF.

Finally, activities with few episodes in a day and with typically clear end and beginning (such as VPA, or, to some extent, screen time) are cognitively easier to estimate than activities that may occur in several short episodes dispersed over the day (such as sedentary time). If the respondent has to make big generalizations (e.g., estimating the total walking or sedentary time), the results are likely to be less useful.

### Strengths and limitations

Strength of our study is that we used objectively measured PA data, and could compare it to subjective data (self-report questionnaires). Our study had also some limitations that need to be recognized. First, we used the data from a project; the questionnaire was adopted from a project, and was not specially designed to our study. A second limitation is that accelerometers GT1M ActiGraph cannot detect some activities, such as cycling, rowing, swimming and upper body resistance training, and may have underestimated PA in the participants in this study therefore some amount of MVPA activities could have been lost during the measurement.

## Conclusions

None of the questionnaires can be used as a reliable estimate of MVPA at an individual level. The boys underreported their MVPA with subjective ratings as compared to objectively assess physical activity. Underreporting was more marked in active boys, whose daily MVPA was at least 60 minutes per day, and was not significant in less active boys. Subjective items referring to specific everyday activities (e.g., screen time) are likely to be more useful than items requiring large generalizations (e.g., sedentary time).

## Supporting Information

S1 Dataset Data used in the current paper. Variables are explained in the first row of the table.
(XLS)
